# Anomalies of a topologically ordered surface

**DOI:** 10.1038/srep10260

**Published:** 2015-06-04

**Authors:** Deepnarayan Biswas, Sangeeta Thakur, Khadiza Ali, Geetha Balakrishnan, Kalobaran Maiti

**Affiliations:** 1 Department of Condensed Matter Physics and Materials’ Science, Tata Institute of Fundamental Research, Homi Bhabha Road, Colaba, Mumbai - 400 005, India; 2 Department of Physics, University of Warwick, Coventry, CV4 7AL, UK

## Abstract

Bulk insulators with strong spin orbit coupling exhibit metallic surface states possessing topological order protected by the time reversal symmetry. However, experiments show vulnerability of topological states to aging and impurities. Different studies show contrasting behavior of the Dirac states along with plethora of anomalies, which has become an outstanding problem in material science. Here, we probe the electronic structure of Bi_2_Se_3_ employing high resolution photoemission spectroscopy and discover the dependence of the behavior of Dirac particles on surface terminations. The Dirac cone apex appears at different binding energies and exhibits contrasting shift on Bi and Se terminated surfaces with complex time dependence emerging from subtle adsorbed oxygen-surface atom interactions. These results uncover the surface states behavior of real systems and the dichotomy of topological and normal surface states important for device fabrication as well as realization of novel physics such as Majorana Fermions, magnetic monopole, etc.

Topological insulators are like ordinary insulators in the bulk with gapless surface states protected by time reversal symmetry^1^,^2^. These materials have drawn much attention in the recent times followed by the proposals of the realization of exotic physics involving Majorana Fermions^3^, magnetic monopoles^4^ etc. In addition to such fundamental interests, the predicted special properties of these states make them useful for the technological applications ranging from spintronics to quantum computations. Although the topological states are protected by time reversal symmetry^5^, numerous experiments show instability of the topological states with aging. Plethora of contrasting scenarios, anomaly on absorption of foreign elements, etc. are observed in the experimental studies^5^,^6^,^7^. It is evident that the real materials are complex and may not be commensurate to the theoretical predictions that makes this issue an outstanding problem in various branches of science and technology.

In order to elucidate these puzzles in real materials, we studied the electronic structure of a typical topological insulator, Bi_2_Se_3_ at different experimental conditions such as the behavior of differently terminated surface, evolution of the electronic structure on aging, etc. employing high resolution photoemission spectroscopy. Bi_2_Se_3_ forms in a layered structure (see Fig. 1(a)) with the quintuple layers of Se-Bi-Se-Bi-Se stacked together by Van der Waals force^8^. The surface electronic structure exhibits topological order with the apex of the Dirac cone, called Dirac Point (DP) at finite binding energies due to finite charge carrier doping arising from impurities, imperfections, etc.^9^,^10^,^11^ These states often show instability with time and complex time evolutions^5^,^6^,^7^, which has been attributed to different phenomena such as relaxation of Van der Waals bond^12^, the surface band bending^5^, dangling surface states^13^, etc. There exists contrasting arguments indicating the necessity of unusually large change in the bond length for the relaxation of Van der Waals bonds^14^. Evidently, the real materials exhibit significant deviations from theoretical wisdom^15^,^16^,^17^ albeit the electronic states being topologically protected. Here, we discover that the behavior of the topological states is dependent on surface terminations. The anomalies in the behavior of the Dirac particles actually depends on subtle interactions of the adsorbates with the surface atoms.

In Fig. 1, we show the signature of Dirac cone representing the topological surface states in the angle resolved photoemission spectroscopic (ARPES) data. The high symmetry points and the Brillouin zone defined in the reciprocal space of Bi_2_Se_3_ are shown in Fig. 1(b). In addition to the metallic Dirac states, several bulk bands cross the Fermi level, 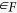 indicating metallicity of the bulk electronic structure^1^,^2^]. Curiously, the Dirac point appears at a significantly high binding energy of about 0.45 eV. To ascertain the reproducibility of these data, the sample was cleaved several times and measured in identical conditions. We discover results of two categories. (i) In one case, DP appears at around 0.3 eV binding energy as shown in Fig. 2, consistent with the earlier results^1^,^2^,^5^,^6^,^7^. We denote this case as ‘Clv1’. (ii) In the other case denoted as ‘Clv2’, the DP appears around 0.45 eV binding energy. Time evolution of the ‘Clv2’ spectra at 20 K is shown in Figs. 1(c)-1(e) and corresponding energy distribution curves (EDCs) in 1(f) - 1(h). Ironically, the DP shifts towards 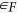 with the increase in time delay from cleaving^7^ suggesting an effective hole doping with time and/or passivation of the electron doped bulk with aging.

DP in the ‘Clv1’ spectra shown in Fig. 2 appears at around 0.3 eV for freshly cleaved surface and gradually shifts away from 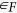 with the increase in time delay suggesting an electron doping with time. Evidently, the contrasting scenario for Clv1 and Clv2 surface is curious. DP appears to stabilize at a long time delay in both cases. The experiments at 200 K exhibit similar trend in energy shift with a slightly different saturation value. All these results indicate that the cleaved surfaces are qualitatively different in the two cases with 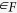 pinned at different energies and anomalous shift of the Dirac point with time. Some element specific study is necessary to reveal the surface chemistry of these materials.

We employed 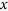-ray photoemission spectroscopy (XPS) to probe the surface chemistry^18^. The normal emission Se 3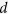 spectrum from Clv2 surface exhibits two peak structures for each spin-orbit split peaks as denoted by ‘A’ (53.1 eV) and ‘B’ (53.4 eV) in Fig. 3(a) for the 3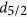 photoemission signal. The center of the 3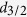 signal appears around 54.1 eV. The feature, A becomes significantly weaker in Clv1 spectra with subsequent enhancement of the feature B. The peak B enhances further in the spectra collected at 60° off-normal emission from Clv1 surface. Off-normal emission makes the technique more surface sensitive^19^,^20^ (the photoemission probing depth, 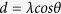, 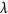 = escape depth and 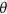 = emission angle with surface normal). The enhancement of B with the increase in surface sensitivity suggests its surface character, thereby, assigning the feature A to the bulk Se photoemission. Thus, the cleaving of Bi_2_Se_3_ leads to different surface terminations exposing Se in Clv1 case and Bi in Clv2 case. We note here that top post removal method was necessary to prepare well ordered clean sample surface exhibiting significantly strong binding between the quintuple layers.

The second important observation is shown in Fig. 3(b), where the 60° angled emission spectra from Clv1 surface exhibit a decrease of B with aging and subsequent increase of two weak features at higher binding energies (marked ‘SeO_*x*_’ in the figure) indicating emergence of a new kind of Se at the cost of some of the surface Se species. The changes in the normal emission spectra from Clv2 surface, however, are not distinct indicating weak influence of aging on the subsurface Se species. The features deriving the Se 3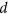 spectra were simulated by a set of asymmetric peaks as shown in Fig. 3(c). The shaded peaks represent the bulk Se photoemission and the other features correspond to the surface Se. The peak position of the emerging Se components with time and their intensity ratio indicate their origin to Se 3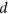 signals from SeO_*x*_ species^21^. Bi 5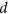 spectra shown in Fig. 3(d), however, exhibit quite similar lineshape at various experimental conditions employed.

Experiments on freshly cleaved surface do not show signature of impurity features. The oxygen 1s signal appears to emerge with aging and gradually grows with the increase in delay time. A representative case is shown in Fig. 4(a) for Clv1 surface after normalizing by the number of scans. The Clv1 spectrum after 6 hours delay exhibits three distinct features denoted by A, B and C in Fig. 4(b). The Clv2 spectrum at about 6 hours delay exhibits relatively more intense A and weaker C with no trace of B suggesting different characteristics of adsorbed oxygens on different surfaces. In both cases, the feature A grows quickly relative to the other features as shown in Fig. 4(c).

Now, the question is, if this surface modification influences Dirac states although they are protected by topological order. In Fig. 4(d), we show the evolution of the Dirac point with aging and observe that the DP in Clv1 spectra appears at around 0.3 eV, on freshly cleaved surface. With increase in time delay, it gradually shifts towards higher binding energies with time and stabilizes around 0.4 eV. The same set of experiments at 200 K exhibit DP around 0.3 eV along with a weaker energy shift saturating around 0.38 eV that opens up new possibilities in understanding the behavior of these exotic states vis a vis existing wisdoms^17^,^22^,^23^. Ironically, the Clv2 spectra exhibit a reverse scenario with DP appearing at much higher binding energy of 0.45 eV and shifting in the opposite direction implying an effective hole doping case. The time evolution of DP can be expressed as,
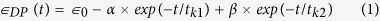


Here, 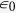 = DP at long delay time, 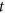. 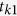 and 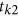 are the time constants. 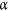 and 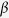 are positive and are related to the electron and hole doping, respectively. The data points at different conditions can be captured remarkably well with the above equation. Now, at 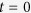, the binding energy at DP is 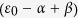. Therefore, the shift of DP can be expressed as
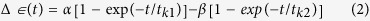
values of 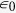, 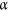 and 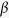 are 0.41, 0.13 & 0.02 for ‘Clv1’ at 20 K; 0.38, 0.12 & 0.02 for ‘Clv1’ at 200 K, and 0.35, 0.16 & 0.25 for ‘Clv2’ at 20 K. The parameters are quite similar in all the cases except a large 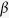 for the ‘Clv2’ case. 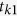 and 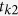 are 6 hrs and 16 hours at 20 K in both the cleaved cases. 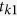 becomes 4 hrs at 200 K leaving 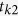 unchanged. The growth of oxygen can be expressed as 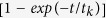 with 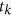 = 16 hrs for the features B & C, and 6 hrs for A at 20 K indicating a possible link between the DP shift and oxygen growth.

Since the time delay primarily modifies the surface, the chemical potential shift must be due to the change in electron count in the surface electronic structure^24^. Three types of oxygen species are found to grow on the surface. The feature A grows quickly and have dominant contribution on Clv2 (Bi terminated) surface. The feature B is absent in Clv2 spectra and have large contribution in Clv1 spectra (Se-terminated surface) suggesting its bonding with surface Se leading to electron doping, while the feature A corresponds to oxygen bonded to Bi leading to hole doping.

The charge densities on the surface are calculated using full potential density functional theory and shown in Fig. 5. The Bi terminated surface exhibit highly extended electron density spreading over a larger spatial distance away from the surface compared to the Se-terminated case. The Fermi surface corresponding to the Bi-terminated surface is significantly larger than that corresponding to Se-terminated case. Thus, DP is expected to appear at higher binding energy in Bi-terminated case compared to the Se-terminated case as found experimentally.

Se and O belong to the same group of the Periodic table with O being the topmost element with higher electronegativity. Therefore, O on Se surface forms SeO_*x*_ complex (signature of SeO_*x*_ appears in the Se 3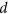 spectra). Se-O bonding will attract electron cloud from the neighborhood reducing the electron density in the Bi-Se neighborhoods as manifested in the left panel of Fig. 5 by increase in spatial charge density contours around oxygen sites relative to the pristine case. Since the conduction band consists of 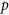 electrons, this would lead to an effective electron doping in the conduction band. On the other hand, oxygen on Bi-terminated surface would form BiO_*x*_ complexes leading to more charge localization in the vicinity of oxygens (see right most panel of Fig. 5) reducing the Fermi surface volume; an effective hole doping scenario.

In summary, we studied the surface electronic structure of a topological insulator, Bi_2_Se_3_ employing high resolution photoemission spectroscopy. We observe the sensitivity of the Dirac states on surface termination. The surface states and the impurities appear to play a complex role leading to complex Fermi surface reconstructions emerging as a shift of the Dirac cone. These materials have been drawing much attention due to their potential technological applications in addition to the fundamental issues of realizing magnetic monopoles, the observation of Majorana fermions, etc. The results presented here reveal the complex microscopic details of the surface states necessary in the realization of such ambitious projects in real materials.

## Method

High quality single crystals of Bi_2_Se_3_ were prepared by Bridgeman method and characterized using 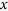-ray Laue diffraction. Angle resolved photoemission (ARPES) measurements were carried out employing Gammadata Scienta R4000 WAL electron analyzer, monochromatic He I and Al 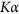 sources with photon energies 21.2 eV and 1486.6 eV, respectively. An open cycle helium cryostat was used to cool down the same. The energy and angle resolution were set to 10 meV and 0.1° for the angle resolved photoemission measurements with monochromatic He I light source to have adequate signal to noise ratio without compromising the resolution necessary for this study. Although the experiment setup could be operated at 300 meV resolution at 1486.6 eV photon energy, 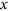-ray photoemission spectroscopic measurements were carried out at 380 meV resolution, which is better than the lifetime broadening of the core level features and enables collection of the spectra with good signal to noise ratio at a relatively short time. The pressure of the experiment chamber during the measurements with the photon sources on was better that 5 × 10^−10^ torr. The sample was cleaved several times *in situ* at the experimental temperature using a top post glued on top of the sample. The well ordered sample surface has been verified by bright sharp low energy electron diffraction spots. 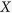-ray photoemission spectra from freshly cleaved sample was found clean with no oxygen or carbon related signals.

The electronic band structure of a slab of Bi_2_Se_3_ was calculated employing *state of the art* full potential linearized augmented plane wave method using Wien2k software^25^. The bulk electronic structure exhibits a gap consistent with its insulating behavior^26^,^27^ and the results from slab calculations show signature of Dirac cone in the presence of spin-orbit coupling.

## Additional Information

**How to cite this article**: Biswas, D. *et al*. Anomalies of a topologically ordered surface. *Sci. Rep.* **5**, 10260; doi: 10.1038/srep10260 (2015).

## Figures and Tables

**Figure 1:**
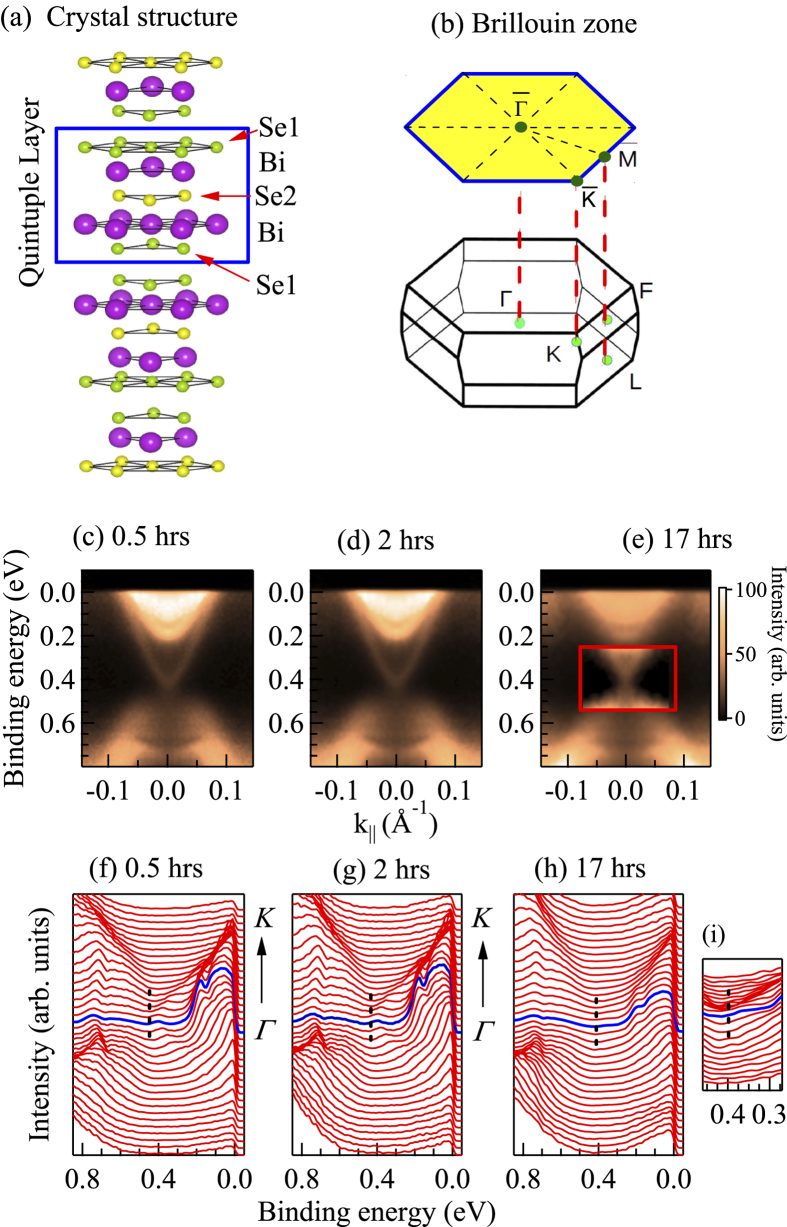
(**a**) Crystal structure of Bi_2_Se_3_ exhibiting stack of quintuple layers of Bi and Se and corresponding (**b**) Brillouin zone. ARPES data at 20 K along 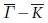 for ‘Clv2’ after (**c**) 0.5 hrs, (**d**) 2 hrs and (**e**) 17 hours of cleaving. (**f**-**g**) show the corresponding energy distribution curves. (**i**) Rescaled spectral region exhibiting the Dirac point with better clarity.

**Figure 2:**
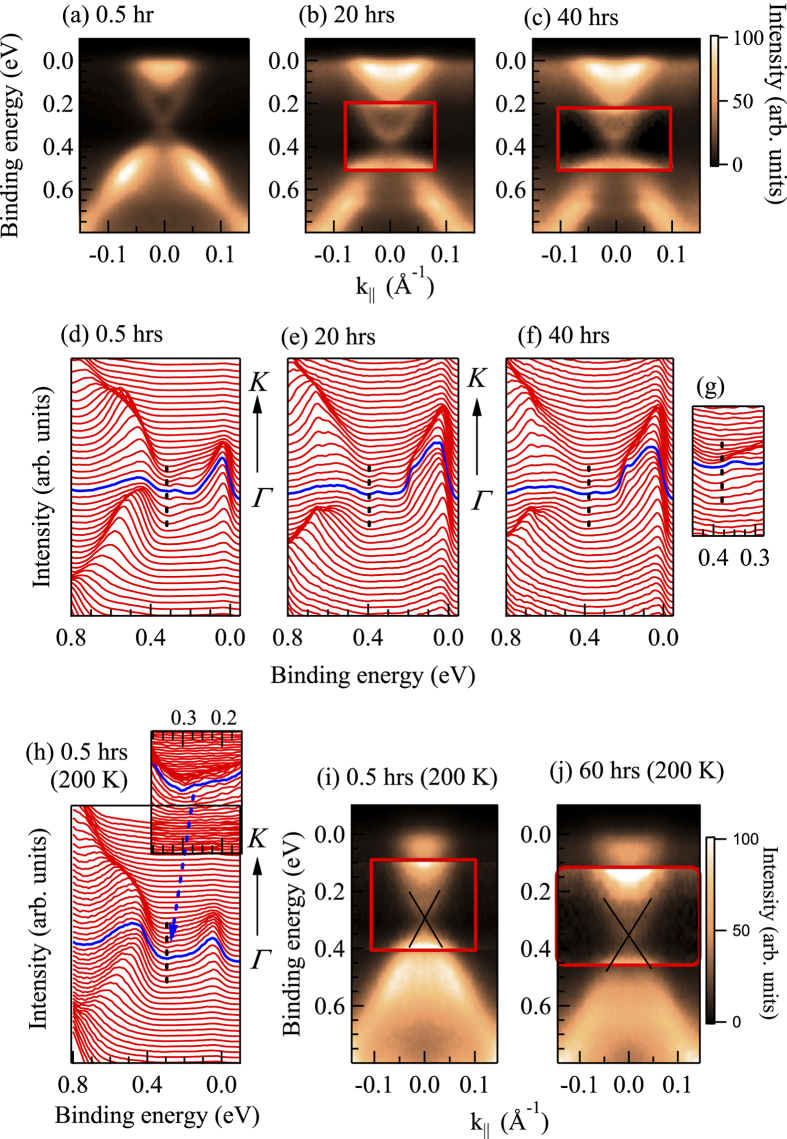
(a) ARPES data at 20 K along 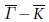 direction from Clv1 surface after (**a**) 0.5 hrs, (**b**) 20 hrs and (**c**) 40 hours after cleaving. (**d**-**f**) show the corresponding energy distribution curves (EDCs). (**g**) Rescaled spectral region near Dirac point. (**h**) EDC at 200 K. ARPES data at 200 K at (**i**) 0.5 hrs and (**j**) 60 hours after cleaving.

**Figure 3:**
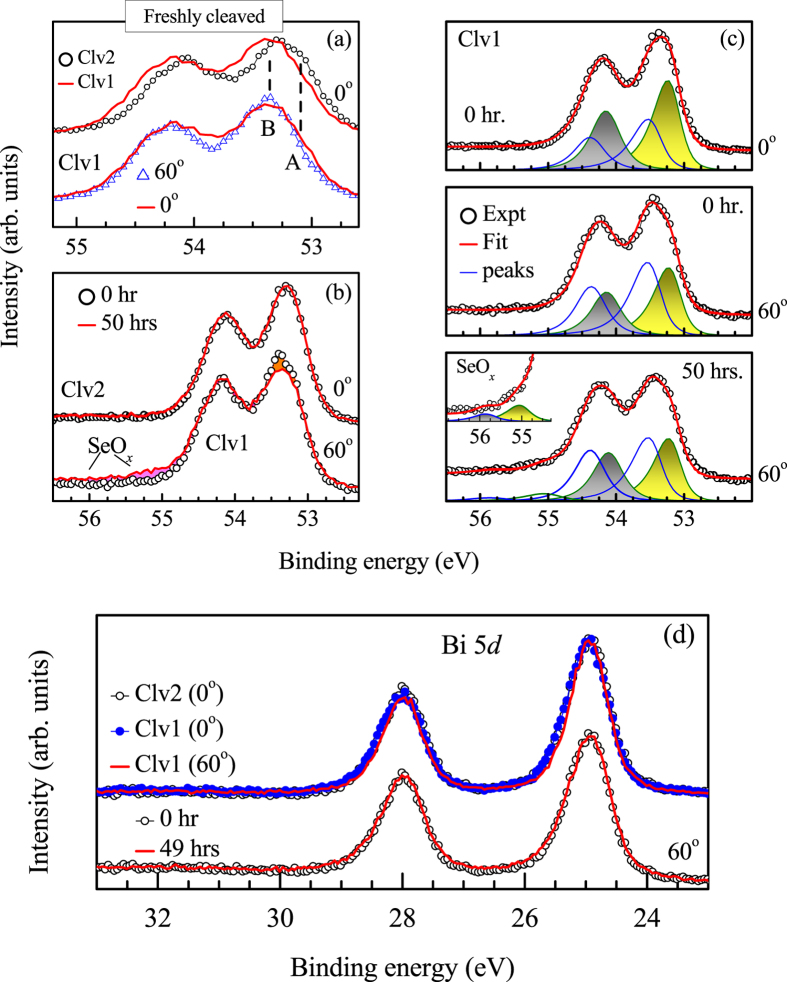
(**a**) Se 3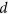 spectra from Clv1 (line) and Clv2 (open circles) surfaces at normal emission. The open triangles are the Clv1 data at 60° off-normal electron emission. (**b**) Se 3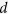 spectra at normal emission from Clv2 surface and at 60° off-normal emission from ‘Clv1’ surface. The data from freshly cleaved surface and after 50 hours of delay are shown by open circles and lines, respectively. (**c**) The fit of the Se 3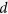 spectra from Clv1 at different emission angles and time delay. (**d**) Bi 4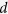 spectra from Clv1 and Clv2 surfaces at different emission angles and delay times exhibiting identical lineshape for every case.

**Figure 4:**
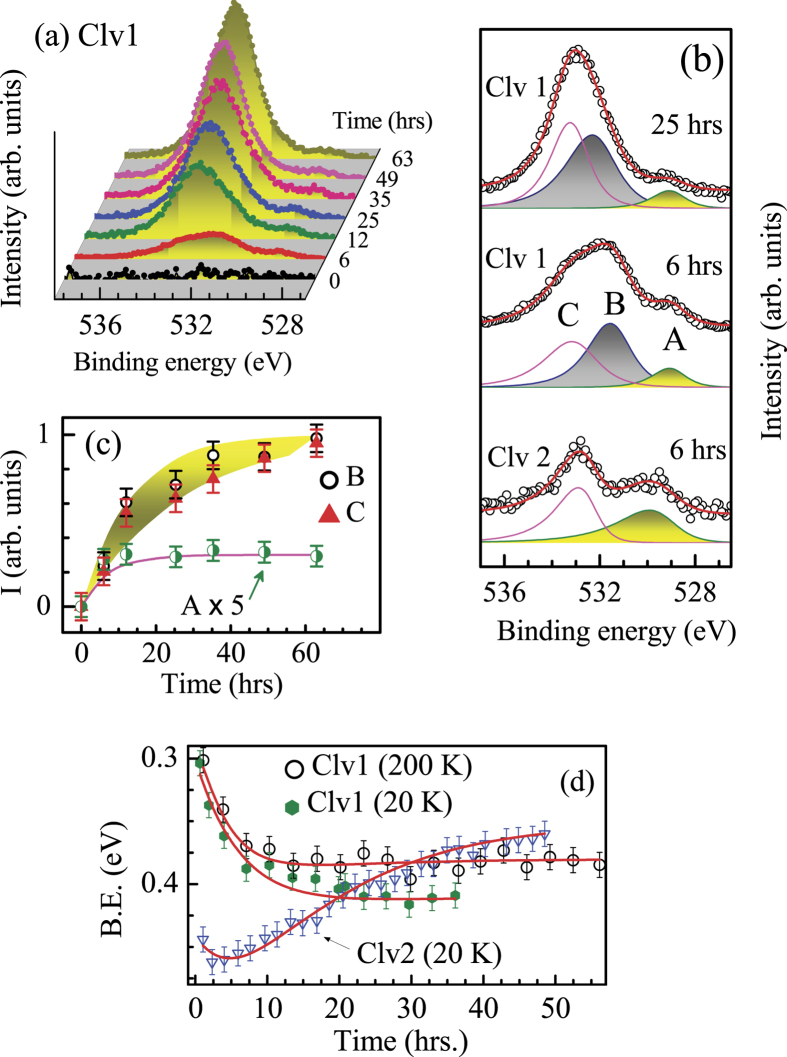
(**a**) Growth of O 1s feature on Clv1 surface with time at 20 K. (**b**) Fit of the O 1s spectra from both Clv1 and Clv2 surfaces with asymmetric peaks. Except the feature A, all the features of 25 hrs delay spectrum are compressed by 2 times for clarity. (**c**) Evolution of the O 1s intensities with time. The line and the shaded region show exponential time dependence. (**d**) Binding energy shift of the Dirac point with time (symbols). The lines show exponential fits.

**Figure 5:**
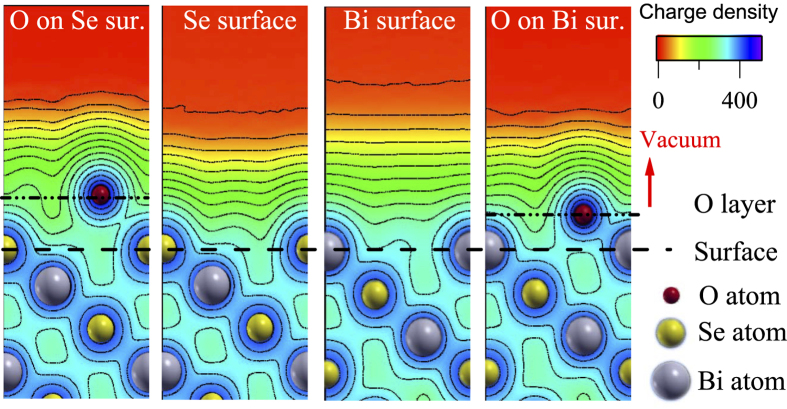
Calculated charge density plots (left to right) for oxygen on Se-terminated surface, Se terminated surface, Bi terminated surface and oxygen on Bi-terminated surface.
